# Surgical Site Infection in Obstetric and Gynecological Surgeries: A Prospective Observational Study

**DOI:** 10.7759/cureus.34855

**Published:** 2023-02-11

**Authors:** Sayali P Kulkarni, Oas Kothari

**Affiliations:** 1 Obstetrics and Gynecology, Sri Aurobindo Institute of Medical Sciences, Indore, IND

**Keywords:** obstetric surgery, gynecological surgery, microorganisms, comorbidities, surgical site infections

## Abstract

Introduction

An infection at an incisional site that develops within 30 days after surgery, or within a year if a prosthetic is implanted, is referred to as a surgical site infection (SSI). They are mainly caused by exogenous and/or endogenous microbes that penetrate the surgical site during surgery (primary infection) or after the procedure (secondary infection). The prevention of SSI should be the ultimate goal of the surgery team and hospital administration.

Methodology

The prospective observational study of SSI consisted of 920 patients who were admitted and underwent surgery between April 2021 and September 2022. After a complete examination, a detailed proforma for the collection of data pertaining to patients in this study was prepared, and patients were included as per the inclusion and exclusion criteria.

Results

The study demonstrated significant results in terms of the association of body mass index (BMI), hemoglobin, and blood sugar level with the SSI status (p<0.05) and nonsignificant results in terms of emergency/elective surgery, type of surgery, and type of incision (p>0.05).

Conclusion

The overall rate of SSI was 9.2% in the present study. The major reasons involved are inadequate infrastructure facilities, different antibiotics policies, and non-uniform pre-, intra-, and post-operative measures that add woes and result in an increased incidence of SSI. In the present study that was undertaken at a teaching and tertiary care center, the SSI incidence is comparatively lower, but with the implementation of correct knowledge and technique, the rate can further be reduced to a large extent.

## Introduction

The second most frequent cause of maternal mortality in obstetrics and gynecology, after postpartum hemorrhage, is infection [1]. Surgical site infections (SSIs), which account for 38% of hospital-acquired infections among surgical patients in the obstetrics and gynecology department, are the most prevalent nosocomial infections. The percentage of SSI varies between 2.5% and 41.9%, according to various reports in the Indian literature [2,3].

An infection at an incisional site that develops within 30 days after surgery, or within a year if a prosthetic is implanted, is referred to as an SSI. Even though this complication is common but nearly rarely fatal, it prolongs the healing of the incision and raises morbidity [4]. Despite recent advances in pathophysiology knowledge and prophylactic measures, SSIs continue to be a major contributor to post-operative morbidity, higher treatment costs, and extended hospital stays [5]. These infections almost never pose a life-threatening hazard, and they have no effect on the overall success of the surgery. However, the patient, her family, and the hospital are troubled by the extended morbidity and extra costs [2,6].

Exogenous and/or endogenous microbes penetrate the surgical site during surgery (primary infection) or after the procedure (secondary infection) [7,8]. Primary infections typically appear five to seven days after surgery and are more dangerous [9]. The majority of SSIs are straightforward infections that just affect the subcutaneous tissue and the skin, although they can occasionally develop into necrotizing infections [10]. Surgical wounds that are infected typically appear with pain, warmth, swelling, pus development, and erythema [11].

The prevention of SSI is the primary responsibility of the surgical team and hospital administration [12]. We have planned to undertake this topic as there is very scanty literature pertaining to surgical site infections in obstetrics and gynecology. Also, within institutions, different units follow their own customized protocols for pre-operative workup, intra-operative techniques, and post-operative care measures to tackle SSI.

This study aimed to ascertain the various factors responsible for the causation of SSI and the proportion of SSI. Hence, the present study was conducted to evaluate the modes and methods to reduce SSI, thereby reducing the cost of treatment, patient dissatisfaction, possible litigation, and prolonged morbidity related to this complication.

## Materials and methods

This prospective observational study of SSI was carried out at Sri Aurobindo Medical College and Postgraduate Institute, Indore, in the obstetrics and gynecology department. Nine hundred twenty patients who were admitted and underwent surgery between April 2021 and September 2022, a span of one-and-a-half years, were included in the study.

The study was initiated after approval by the Institutional Ethics Committee (IEC) and Scientific Research Committee of Sri Aurobindo Institute of Medical Sciences, Indore, with IEC number SAIMS/RC/IEC/2021/54. After admitting the patients and taking proper history related to past, medical and surgical, clinical examination including systemic examination, along with vital parameters, a provisional diagnosis was made.

After conducting all required pathological and radiological interventions, a confirmatory diagnosis of the patient was made, and the required intervention was decided. After confirming the required surgical intervention and mode of surgery, pre-operative investigations were sent, and a pre-anesthetic checkup was done.

A detailed proforma for the collection of data pertaining to patients in this study was prepared, and patients were included as per the inclusion and exclusion criteria. Patients undergoing elective or emergency obstetric or gynecological abdominal surgery and patients with SSI occurring within 30 days after the operation were involved in the study. Patients not willing to participate, patients below 18 years and above 60 years, patients who underwent re-exploration surgery, and patients who were operated on elsewhere and were referred for SSI or any other reason were not involved in the study.

Broad measures that were followed in all the patients involved the following: skin preparation was done by removing the hairs by shaving with a razor immediately before the operation in emergency cases. In elective cases, skin preparation was done the night before by shaving, and all patients were given pre-operative intravenous antimicrobial prophylaxis one hour to four hours before the surgery in the recovery room. A third-generation cephalosporin was usually employed, followed by pre-operative skin preparation done with Savlon, alcohol, and povidone-iodine; care was taken to achieve complete hemostasis at the site of operation with electrocautery. In all cases, povidone-iodine was applied before applying skin sutures; in obese patients, either the subcutaneous fat was approximated with interrupted absorbable sutures or a subcutaneous drain was kept, and abdominal drains were used whenever needed.

Post-operatively, the patient's wound was examined in all the cases by an assigned resident surgeon, and the findings were recorded on the case sheet. The initial post-operative inspection of the wound was performed after 72 hours or even sooner if there was soakage, there was discomfort at the wound site, or there is high body temperature. Thereafter, it was performed on the 10th day or as and when necessary. Patients were followed up for 10 days in case of subcuticular sutures and until suture removal in mattress suture. In case of the presence of signs of surgical site infection, swab was sent for culture and sensitivity.

Patients were informed of the symptoms of SSI and advised to notify the observer right away after seeing the first SSI symptom for a month. The discharged patients were advised for ongoing follow-up care for a month in the outpatient department. Every time a patient was seen during the visit, a thorough examination was performed, and any infections were quickly identified and reported.

## Results

The distribution of a total of 920 patients on the basis of age group demonstrated a higher proportion of 65.1% for 18-40 years and a lower proportion of 34.9% for age group 41-60 years. Table 1 demonstrated patient classification on the basis of SSI status. The higher proportion of 90.8% was absent, and the lower proportion of 9.2% was present.

**Table 1:** Distribution on the basis of SSI status. SSI: surgical site infection

SSI status	Frequency	Percent
Absent	835	90.8
Present	85	9.2
Total	920	100.0

According to the distribution on the basis of BMI, the higher proportion of 82.7% was for 25-30 kg/m^2^, and the lower proportion of 17.3% was for 18-24.9 kg/m^2^. Table 2 demonstrates the association between body mass index (BMI) range and SSI status.

**Table 2:** Association between BMI range and SSI status. BMI, body mass index; SSI, surgical site infection; df, degrees of freedom

BMI (kg/m^2^)		SSI status		Total
		Absent	Present	
18-24.9	Count	136	23	159
	%	85.5%	14.5%	100.0%
25-30	Count	699	62	761
	%	91.9%	8.1%	100.0%
Total	Count	835	85	920
	%	90.8%	9.2%	100.0%
Pearson chi-square	Value	df	P-value	Result
	6.261a	1	0.012	Significant

According to the distribution of patients on the basis of emergency/elective, the higher proportion of 55.3% was for emergency, and the lower proportion of 44.7% was for elective. Table 3 demonstrates the association between emergency/elective and SSI status.

**Table 3:** Association between emergency/elective and SSI status. SSI, surgical site infection; df, degrees of freedom

Emergency/elective		SSI status		Total
		Absent	Present	
Elective	Count	373	38	411
	%	90.8%	9.2%	100.0%
Emergency	Count	462	47	509
	%	90.8%	9.2%	100.0%
Total	Count	835	85	920
	%	90.8%	9.2%	100.0%
Pearson chi-square	Value	df	P-value	Result
	0.000a	1	0.995	Nonsignificant

According to the type of surgery performed, a higher proportion of 55.7% was for obstetric surgery, and the proportion of 44.3% was for gynecological surgery. Table 4 demonstrates the association between the type of surgery and SSI status.

**Table 4:** Association between type of surgery and SSI status. SSI, surgical site infection; df, degrees of freedom

Type of surgery		SSI status		Total
		Absent	Present	
Gynecological surgery	Count	363	45	408
	%	89.0%	11.0%	100.0%
Obstetric surgery	Count	472	40	512
	%	92.2%	7.8%	100.0%
Total	Count	835	85	920
	%	90.8%	9.2%	100.0%
Pearson chi-square	Value	df	P-value	Result
	2.802a	1	0.094	Nonsignificant

According to the type of incision, a higher proportion of 55.7% was for Pfannenstiel, and the lower proportion of 8.9% was for midline vertical. Table 5 demonstrates the association between the type of incision and SSI status.

**Table 5:** Association between the type of incision and SSI status. SSI, surgical site infection; df, degrees of freedom

Type of incision		SSI status		Total
		Absent	Present	
Laparoscopic port	Count	83	7	90
	%	92.2%	7.8%	100.0%
Low transverse incision	Count	205	31	236
	%	86.9%	13.1%	100.0%
Midline vertical	Count	75	7	82
	%	91.5%	8.5%	100.0%
Pfannenstiel incision	Count	472	40	512
	%	92.2%	7.8%	100.0%
Total	Count	835	85	920
	%	90.8%	9.2%	100.0%
Pearson chi-square	Value	df	P-value	Result
	5.793a	3	0.122	Nonsignificant

According to the basis of hemoglobin, a higher proportion of 58.0% was for 7-11, and the lower proportion of 1.5% was for <7. Table 6 demonstrates the association between the basis of hemoglobin and SSI status.

**Table 6:** Association between the basis of hemoglobin and SSI status. SSI, surgical site infection; df, degrees of freedom

Hemoglobin (g/dL)		SSI status		Total
		Absent	Present	
<7	Count	0	14	14
	%	0.0%	100.0%	100.0%
7-11	Count	477	57	534
	%	89.3%	10.7%	100.0%
>11	Count	358	14	372
	%	96.2%	3.8%	100.0%
Total	Count	835	85	920
	%	90.8%	9.2%	100.0%
Pearson chi-square	Value	df	P-value	Result
	152.142a	2	0.000	Significant

Table 7 demonstrates the association between the basis of random blood sugar level and SSI status.

**Table 7:** Association between random blood sugar level and SSI status. SSI, surgical site infections; RBS, random blood sugar; df, degrees of freedom

RBS level		SSI status		Total
		Absent	Present	
<140	Count	662	34	696
	%	95.1%	4.9%	100.0%
≥140	Count	173	51	224
	%	77.2%	22.8%	100.0%
Total	Count	835	85	920
	%	90.8%	9.2%	100.0%
Pearson chi-square	Value	df	P-value	Result
	64.627a	1	0.000	Significant

In maximum cases, no growth was detected second; the higher proportion of 30.59% was for *E. coli* organism, and the lower proportion of 7.06% was for methicillin-resistant *Staphylococcus aureus* (MRSA). Table 8 describes the distribution of patients according to the organisms detected.

**Table 8:** Distribution on the basis of organisms detected. MRSA: methicillin-resistant *Staphylococcus aureus*

Organism detected	Frequency	Percent
E. coli	26	30.59
Klebsiella pneumoniae	9	10.59
MRSA	6	7.06
Others	16	18.82
No growth	28	32.94
Total	85	100.0

## Discussion

From April 2021 to September 2022, 920 patients who underwent abdominal surgery at the obstetrics and gynecology department of Sri Aurobindo Medical College and Postgraduate Institute, Indore, participated in this prospective observational study.

Out of 920 patients, a higher proportion of 65.1% for 18-40 years and a lower proportion of 34.9% for the age group 41-60 years were seen. Additionally, out of 920 patients, SSI was absent in 835 (90.8%) patients and was present in 85 (9.2%) patients. These figures indicate that the chances of SSIs with age have no correlation; this is contradictory to many studies that have shown that the chances of SSI increase with advancing age, which may be attributed due to various factors in the present study, i.e., patients beyond the age of 60 not included.

Age greater than 45 years was revealed to be a risk factor for getting SSI by Suchitra and Lakshmidevi [13] In a related study, Mawalla et al. found that the incidence of SSI varied by age group and was as follows: out of 61 patients, 12 (19.6%) got SSI. Twenty-one (30.8%) out of 68 patients in the age group of 41-60 years had SSI, while 14 had SSI out of 44 (31.8%) patients beyond the age of 60. Out of 77 patients in the age group 21-40 years, 23.3% had SSI [14]. Similar findings of an increase in SSI incidence with advancing age were found in research conducted in India by Kamat et al. [15].

The overall SSI incidence in the present study was 9.2%, which is comparable to various reports in the Indian literature, that is, 2.5%-41.9% [6,16,17]. Compared to rates in the United States of America and other European nations, the incidence rate is significantly greater [5]. Hospitals in Asia have a greater prevalence of SSI, which is a reflection of the region's subpar awareness of related infections and infection control procedures.

Eighty-five patients had SSI, and of those, 62 (72.94%) had a body mass index (BMI) between 25 and 30 kg/m^2^, whereas 23 (27.06%) had a BMI between 18 and 25 kg/m^2^. These data demonstrate that individuals with a BMI of more than 25 kg/m^2^ had a higher infection rate. BMI>35 kg/m^2^ has been identified by Awan et al. [18] as a risk factor for SSI. A BMI of more than 27 kg/m^2^ was listed as a risk factor for SSI in the study by Kurmann et al. [19]. In line with earlier studies on SSI, it was found in the current study that having a higher BMI increases the risk of developing SSI.

Thirty-one individuals (9.25%) with SSI were among the 411 patients who underwent elective surgery. Forty-seven (9.23%) of the 599 emergency surgery patients had SSI. This contradicts numerous studies that indicate increased risks of SSI in emergency procedures by revealing no variation in the likelihood of SSI in emergency versus elective surgeries. In emergency procedures, the incidence of SSI was 39.3%, while in routine surgeries, it was 22.4% [17]. According to Mawalla et al., the incidence of SSI is 20% in normal cases and 27% in emergency cases [14].

Out of 920 total patients, 408 underwent gynecological surgery, and 512 underwent obstetric surgery, of which 45 (11.03%) and 40 (7.8%) had SSI, respectively. Out of 408 gynecological surgeries, 240 was total abdominal hysterectomy, of which 30 had SSI; 62 was laparoscopic hysterectomy, of which four had SSI; 30 was exploratory laparotomy, of which seven had SSI; 42 was staging laparotomy, of which only one had SSI; 26 was another laparoscopic surgery, of which three had SSI; two was laparoscopic tubal ligation, of which none had SSI; and six was conventional tubal ligation, of which none had SSI.

Fourteen patients in our study had levels of hemoglobin of <7 g/dL, and notably, all had SSI. Five hundred thirty-four patients had hemoglobin between 7 and 11 g/dL, out of which 57 (10.7%) had SSI. Three hundred seventy-two patients had hemoglobin of >11 g/dL, out of which 14 (3.8%) had SSI. Wound infections were higher in patients operated on with hemoglobin of less than 11 g/dL. Statistical analysis shows chi-square of 152, and p-value is less than 0.0001, which was significant; this shows that there was a correlation between hemoglobin and SSI.

Without certainty, patients who have low hemoglobin have a harder time fighting infection. In their study from 2001, Malone et al. demonstrated that anemia, both pre-operative and post-operative, was linked to an increased risk of wound infection. Our observations are consistent with other research on the topic because post-operative anemia was present in 91% of the patients with SSI in their series [20].

Since many years ago, it has been understood that diabetic surgery patients are more likely to experience severe side effects such as poor wound healing, wound infections, cardiac compromise, and mortality. In our study, 696 patients had blood sugar levels less than or equal to 140 mg/dL, out of which 34 (4.8%) had SSI. Two hundred twenty-four patients had blood sugar levels of more than 140 mg/dL, out of which 51 (22.77%) had SSI. Statistical analysis shows chi-square of 64.627 and a p-value of <0.002, which was significant; this shows that there was a correlation between RBS and SSI, and this shows that patients with more sugar levels had an increased chance of getting an infection. A retrospective analysis of 995 patients who underwent general and vascular surgery found that for every 40% rise in blood glucose above 110 mg/dL, the prevalence of post-operative infection rose by 30% [21]. Pre-operative fasting blood glucose level (FBGL) was discovered by Kamat et al. to be a statistically significant variable linked with SSI at a tertiary hospital in Goa. According to Kamat et al., the mean FBGL was 153 mg/dL in SSI patients and 115 mg/dL in non-SSI patients [15]. Thus, the current study is comparable [11,22]. Close observation is necessary, and the patients are advised to keep the maximal glucose aim below 180 mg/dL [21].

In the current investigation, *E. coli* was identified more frequently (30.5%), and the majority of cases showed no growth (32.94%); this incidence was followed by *Klebsiella pneumoniae* (10.59%). *Staphylococcus aureus* was identified in numerous research as the most often isolated organism, but the observation was in contrast to various studies [6,18]. Similar research was conducted by Sahu et al. in 2008 on 200 patients, in which *E. coli* was the most frequently recovered bacterium [23]. In the research by Giri et al. on 230 patients, the infection rate was 23%, with *E. coli* accounting for the majority of the organisms (43.4%) [24].

As the important purpose was to study the rate of SSI in this institution, other parameters representing all the abdominal surgical procedures including the severity of SSI, resulting economical losses, and disfigurement could not be studied probably because of the lack of time and resources. The second objective involves the type of surgery, for example, gynecological surgery or obstetric surgery, that has a deleterious effect on the healing of the wound, which leads to SSI. Furthermore, underlying diseases such as diabetes, anemia, and obesity affect or impair immune function, which raises the risk of SSI. They are significant contributors to rising pre-operative hospital stays, which in turn raise the rate of SSI.

Inadequate infrastructure facilities, different antibiotics policies, and non-uniform pre-, intra-, and post-operative measures add woes and result in an increased incidence of SSI. In the present study that was undertaken at a teaching and tertiary care center, the SSI incidence is comparatively lower, but with the implementation of correct knowledge and technique, the rate can further be reduced to a large extent. More identical studies are needed by healthcare providers to recognize the local factor so that they can be rectified and the morbidity associated with SSI can be decreased.

## Conclusions

In the present study, the overall rate of SSI was 9.2%. In the present institute where the current study was undertaken, *E. coli* was the most commonly isolated organism with SSI. Very few studies are available on this topic in Indian literature, and the available studies show very high incidences of SSI in our country. This may be attributed to lower socioeconomic and nutritional status and the presence of numerous comorbidities. In the present study that was undertaken at a teaching and tertiary care center, the SSI incidence is comparatively lower, but with the implementation of correct knowledge and technique, the rate can further be reduced to a large extent.
